# Advancing health equity: evaluating AI translations of kidney donor information for Spanish speakers

**DOI:** 10.3389/fpubh.2025.1484790

**Published:** 2025-01-27

**Authors:** Oscar A. Garcia Valencia, Charat Thongprayoon, Caroline C. Jadlowiec, Shennen A. Mao, Napat Leeaphorn, Pooja Budhiraja, Nadeen Khoury, Justin H. Pham, Iasmina M. Craici, Maria L. Gonzalez Suarez, Wisit Cheungpasitporn

**Affiliations:** ^1^Division of Nephrology and Hypertension, Department of Medicine, Mayo Clinic, Rochester, MN, United States; ^2^Division of Transplant Surgery, Department of Surgery, Mayo Clinic, Phoenix, AZ, United States; ^3^Department of Transplant Surgery, Mayo Clinic, Jacksonville, FL, United States; ^4^Division of Nephrology and Hypertension, Department of Medicine, Mayo Clinic, Phoenix, AZ, United States; ^5^Division of Nephrology, Department of Medicine, Henry Ford Hospital, Detroit, MI, United States

**Keywords:** health equity, artificial intelligence, language translation models, cultural competency, living kidney donation, Spanish-speaking populations, healthcare communication barriers, ChatGPT

## Abstract

**Background:**

Health equity and access to essential medical information remain significant challenges, especially for the Spanish-speaking Hispanic population, which faces barriers in accessing living kidney donation opportunities. ChatGPT, an AI language model with sophisticated natural language processing capabilities, has been identified as a promising tool for translating critical health information into Spanish. This study aims to assess ChatGPT’s translation efficacy to ensure the information provided is accurate and culturally relevant.

**Methods:**

**T**his study utilized ChatGPT versions 3.5 and 4.0 to translate 27 frequently asked questions (FAQs) from English to Spanish, sourced from Donate Life America’s website. The translated content was reviewed by native Spanish-speaking nephrologists using a standard rubric scale (1–5). The assessment focused on linguistic accuracy and cultural sensitivity, emphasizing retention of the original message, appropriate vocabulary and grammar, and cultural relevance.

**Results:**

The mean linguistic accuracy scores were 4.89 ± 0.32 for GPT-3.5 and 5.00 ± 0.00 for GPT-4.0 (*p* = 0.08). The percentage of excellent-quality translations (score = 5) in linguistic accuracy was 89% for GPT-3.5 and 100% for GPT-4.0 (*p* = 0.24). The mean cultural sensitivity scores were 4.89 ± 0.32 for both GPT-3.5 and GPT-4.0 (*p* = 1.00). Similarly, excellent-quality translations in cultural sensitivity were achieved in 89% of cases for both versions (*p* = 1.00).

**Conclusion:**

ChatGPT 4.0 demonstrates strong potential to enhance health equity by improving Spanish-speaking Hispanic patients’ access to LKD information through accurate and culturally sensitive translations. These findings highlight the role of AI in mitigating healthcare disparities and underscore the need for integrating AI-driven tools into healthcare systems. Future efforts should focus on developing accessible platforms and establishing guidelines to maximize AI’s impact on equitable healthcare delivery and patient education.

## Introduction

The quest for health equity and accessible medical information remains a critical and ongoing challenge in modern healthcare. This challenge is particularly pronounced among minority populations, such as the Spanish-speaking Hispanic community, who often encounter substantial barriers in accessing vital health services and information (1–3). Recent studies emphasize that language barriers disproportionately affect Spanish-speaking patients, leading to disparities in access to living kidney donation (LKD) information and transplant opportunities (3–5). Language-concordant materials and culturally tailored communication strategies have been identified as critical for addressing these disparities, particularly for populations with low health literacy (6–10). Among these barriers is the limited availability of culturally sensitive and linguistically appropriate medical information, particularly concerning LKD (11–14).

LKD plays a critical role in the management of patients with end-stage kidney disease, offering significant benefits such as improved survival rates and enhanced quality of life compared to long-term dialysis (15, 16). While dialysis remains an important treatment option for many patients, LKD provides unique advantages, particularly for those seeking to avoid the long-term complications associated with dialysis. The success of LKD programs hinges on effective communication and the availability of detailed, accurate, and culturally relevant information for potential donors and recipients (4, 17–21). Ensuring equitable access to such information is vital to bridging disparities in transplant opportunities and improving outcomes for diverse patient populations. Studies conducted over the past decade highlight the need for improved accessibility to such information, as language barriers continue to hinder Spanish-speaking patients’ understanding of LKD processes and their participation in transplant programs (5, 16, 22). For example, efforts to expand outreach in Hispanic communities underscore the importance of addressing both linguistic and cultural dimensions in healthcare delivery (23–26). Additionally, systemic challenges, such as inadequate interpreter services and the lack of language-specific digital tools, further exacerbate these inequities (27–30). This reality underscores the critical need to bridge the information gap and ensure that all individuals, regardless of language or cultural background, have equal access to healthcare information and opportunities (31–33). Failure to address these disparities can lead to poorer health outcomes and a perpetuation of systemic inequalities in healthcare access and quality (31–34).

In this context, artificial intelligence (AI), particularly advanced language models like ChatGPT, offers a promising solution to this problem. With its sophisticated natural language processing capabilities, ChatGPT can play a pivotal role in translating critical health information into Spanish, thereby enhancing the accessibility and understanding of such information for the Hispanic community (35, 36). Beyond translation, ChatGPT holds significant potential in CKD and transplant care by simplifying complex medical terminology (37), empowering patients with clear explanations of their condition, treatment options, and the transplant process (38, 39). It can act as a conversational agent, addressing patient queries in real-time and reducing reliance on interpreters. Additionally, ChatGPT’s ability to deliver culturally tailored education materials enhances patient engagement (40), while its role in pre-transplant education—providing customized content and streamlining informed consent—demonstrates its capacity to address healthcare communication barriers and promote health equity (34). Recent advancements in AI have demonstrated its potential to improve health equity by providing accurate translations and culturally relevant materials (29, 41). For instance, AI-driven applications have been successfully utilized to address gaps in patient education, facilitate informed consent, and support healthcare navigation for Spanish-speaking patients (40, 42, 43). The AI’s ability to comprehend and generate human-like text in multiple languages makes it a potentially invaluable tool in breaking down language barriers in healthcare communication (34, 36). However, the efficacy of ChatGPT in providing accurate and culturally sensitive translations is an area that requires thorough investigation (44–46).

This study aims to assess the effectiveness of ChatGPT versions 3.5 and 4.0 in translating essential information about LKD from English to Spanish. The evaluation of these translations is critical to ensuring that the information provided is not only linguistically accurate but also culturally relevant and sensitive to the specific aspects of Hispanic culture. The importance of this study lies not only in its potential to improve access to health information for the Spanish-speaking Hispanic community but also in its broader implications for health equity and the reduction of healthcare disparities. By demonstrating the effectiveness of AI tools like ChatGPT in breaking down language barriers, the study contributes to the ongoing efforts to reduce healthcare disparities and promote inclusivity in medical communication. Additionally, the findings of this study have significant implications for the integration of AI in healthcare, particularly in developing accessible platforms for patient education and consent.

## Methods

### Data collection

The study identified 27 frequently asked questions (FAQs) (Online supplementary data) related to LKD from Donate Life America’s website (47). These questions were chosen due to their relevance and importance in the context of kidney donation, covering a broad spectrum of topics necessary for patient education and informed decision-making (48). The selection criteria for these FAQs emphasized their prevalence in patient inquiries and their significance in the overall understanding of the kidney donation process (48).

### AI language model usage

Upon the collection of the FAQs, the study utilized two versions of the AI chatbot, ChatGPT - versions 3.5 and 4.0 (49). These chatbots were employed to translate the English text of the FAQs into Spanish. The choice of these particular versions of ChatGPT was based on their advanced natural language processing capabilities and their potential for producing accurate and contextually relevant translations. Each question was inputted into both versions of the language model separately, and the outputs were collected for further evaluation.

### Systematic evaluation of the translations

To assess the quality of the translations, a standard rubric scale (Online supplementary data) was developed and applied (50). This scale ranged from 1 to 5, with 1 indicating poor quality and 5 representing excellent quality. The evaluation criteria encompassed several key aspects:

1. Accuracy: Assessing the linguistic precision and the retention of the original message’s meaning.
2. Cultural Sensitivity: Evaluating the appropriateness of vocabulary, grammar, and expressions in the context of Hispanic culture.

Cultural sensitivity, a key focus of this study, was defined as the ability of the translation to effectively convey the intended meaning while respecting and aligning with the cultural norms, values, and linguistic characteristics of Spanish-speaking Hispanic populations. This definition was informed by established frameworks in cross-cultural healthcare communication and translation studies, including principles outlined by Elder et al. (2009) (6) on health communication in Latino communities and guidance from Madden (2015) (7) on cultural health capital.

To assess the quality of the translations, a standard rubric scale was developed and applied, encompassing linguistic accuracy and cultural sensitivity. The evaluation was conducted by two native Spanish-speaking nephrologists, both of whom are Mexican and have extensive clinical experience treating Hispanic populations in the United States and Mexico. Their shared cultural background provided a strong basis for evaluating the linguistic accuracy and cultural relevance of the translations, particularly in the context of Mexican Spanish, which represents one of the most widely spoken variants of the language among Spanish-speaking populations in North America.

To assess cultural appropriateness, the study employed native Spanish-speaking nephrologists who possess not only linguistic proficiency but also a deep understanding of the cultural aspects relevant to the Hispanic community. Participants were provided with explicit instructions to evaluate each translation for its alignment with cultural norms, sensitivity to potentially sensitive topics, and appropriateness of vocabulary and expressions. A standardized rubric was used to score cultural sensitivity, ensuring a consistent and structured assessment process. The rubric included criteria such as the use of culturally relevant terminology, avoidance of culturally inappropriate phrases, and the retention of cultural context within the translations. By grounding the evaluation in these established criteria and frameworks, the study ensured a robust and comprehensive assessment of the cultural appropriateness of the AI-generated translations.

Each translated FAQ was independently reviewed and scored by native Spanish-speaking nephrologists, who possess expertise in both the language and the medical field of nephrology. This approach ensured a comprehensive and expert assessment of the translations, considering both linguistic and medical accuracy.

### Statistical analysis

The mean scores for linguistic accuracy and culture sensitivity were summarized as mean ± standard deviation (SD). The difference in mean score between GPT-3.5 and 4 was tested using Student’s t test. The excellent quality translation (score = 5) in term of linguistic accuracy and culture sensitivity were summarized as number (percentage). The difference in percentage of excellent translation between GPT-3.5 and 4 was tested using Fisher’s exact test. The two-tailed *p*-value less than 0.05 was considered statistically significant. Statistical analyses were performed using JMP statistical software (version 17, SAS Institute, Cary, NC).

## Results

**Figure 1:**
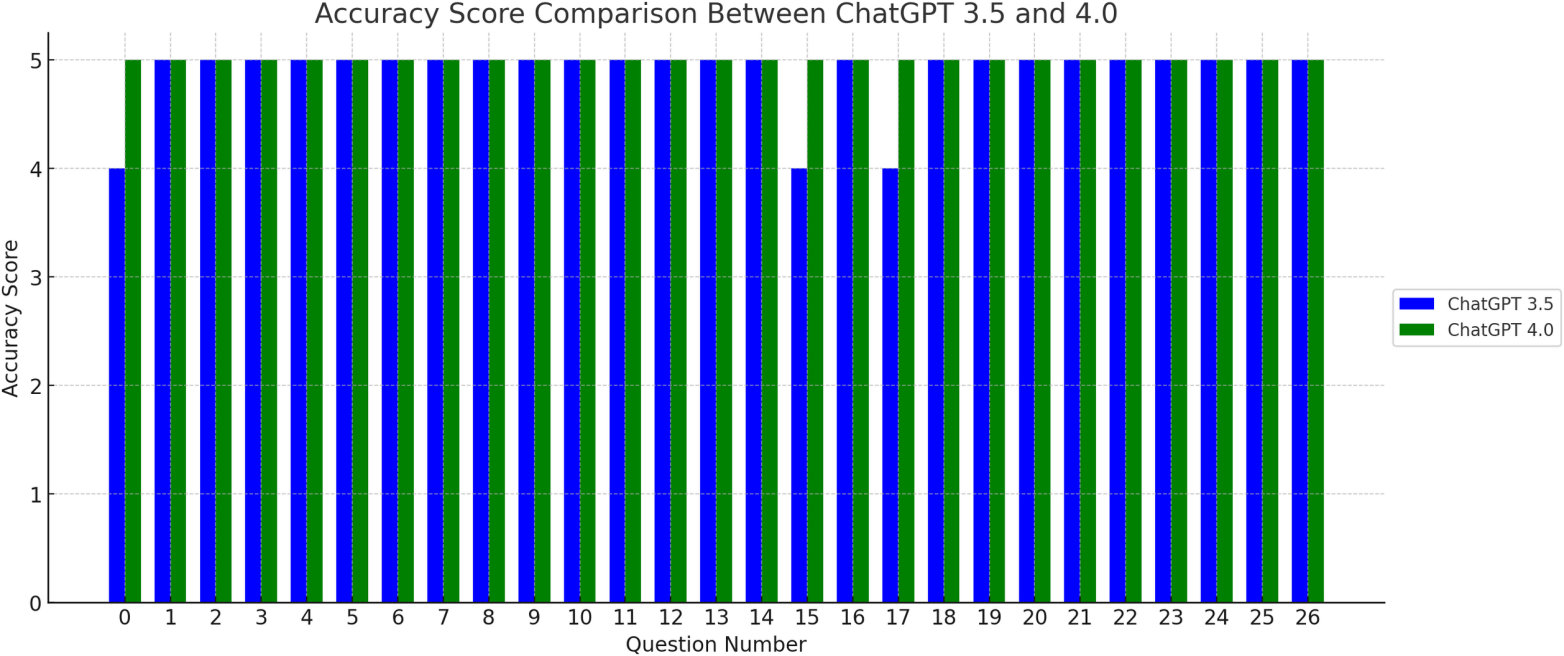
Accuracy Score Comparison Between ChatGPT 3.5 and 4.0. The bar graph above illustrates the accuracy scores for each question, comparing the performance of ChatGPT versions 3.5 and 4.0. The x-axis indicates the question number, while the y-axis represents the accuracy score.

The score for linguistic accuracy and cultural sensitivity of GPT-3.5 and GPT-4.0 for individual FAQs, shown in Supplementary Table S1, was either 4 or 5. The mean linguistic accuracy score was 4.89 ± 0.32 for GPT-3.5 and 5.00 ± 0.00 for GPT-4, Figure 1. There was no significant difference in mean linguistic accuracy score between GPT-3.5 and 4 (*p* = 0.08). The percentage of excellent quality translation in term of linguistic accuracy was 89% for GPT-3.5 and 100% for GPT-4. There was no significant difference in excellent quality translation in term of linguistic accuracy between GPT-3.5 and 4 (*p* = 0.24).

**Figure 2:**
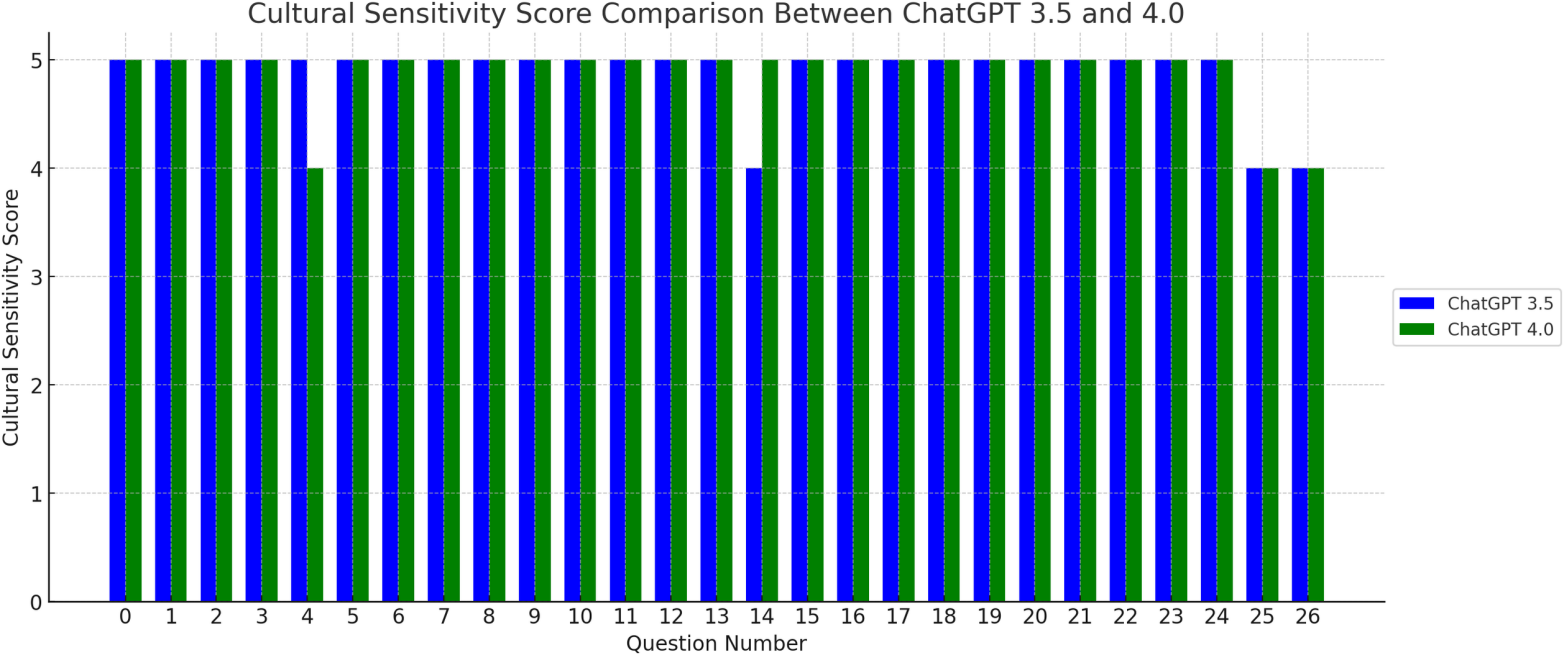
Cultural sensitive score comparison between ChatGPT 3.5 and 4.0. The bar graph here displays the cultural sensitivity scores for each translated question, comparing ChatGPT versions 3.5 and 4.0. Similar to the accuracy score graph, the x-axis represents the question number, while the y-axis shows the cultural sensitivity score.

The mean cultural sensitivity score was 4.89 ± 0.32 for GPT-3.5 and 4.89 ± 0.32 for GPT-4.0, Figure 2. There was no difference in mean culture sensitivity score between GPT-3.5 and 4 (*p* = 1.00), Table 1. The percentage of excellent quality translation in term of culture sensitivity was 89% for both GPT-3.5 and 4. There was no significant difference in excellent quality translation in term of culture sensitivity between GPT-3.5 and 4 (*p* = 1.00).

**Table 1:** The mean score for linguistic accuracy and culture sensitivity of GPT-3.5 and 4.0.

	Linguistic accuracy			Culture sensitivity		
	GPT-3.5	GPT-4.0	*p*-value	GPT-3.5	GPT-4.0	*p*-value
Score, mean ± SD	4.89 ± 0.32	5.00 ± 0.00	0.08	4.89 ± 0.32	4.89 ± 0.32	1.00
Excellent quality, *n* (%)	24 (89%)	27 (100%)	0.24	24 (89%)	24 (89%)	1.00

## Discussion

The pursuit of health equity and accessible medical information is a fundamental challenge in contemporary healthcare, especially pronounced among minority populations such as the Spanish-speaking Hispanic community (51, 52). These groups frequently encounter substantial barriers in accessing essential health services and information, aggravated by a scarcity of culturally sensitive and linguistically appropriate medical resources (6–8, 52). This shortfall is particularly acute in areas like LKD, a critical element of healthcare providing life-saving solutions for individuals with end-stage kidney disease (16, 53). The effectiveness of these programs heavily relies on effective communication and the availability of detailed, accurate, and culturally relevant information for potential donors and recipients. However, the Spanish-speaking Hispanic population in the United States often faces difficulties in accessing such information due to language barriers and cultural differences (54). This situation highlights an urgent need to bridge this information gap, ensuring that all individuals, regardless of their language or cultural background, have equal access to healthcare information and opportunities (55).

This study demonstrates several notable strengths in its research design and execution. The involvement of native Spanish-speaking nephrologists as expert evaluators ensures that both linguistic accuracy and cultural sensitivity are assessed by individuals with a deep understanding of the language and medical context. Utilizing a clear, standardized 5-point rubric further strengthens the reliability of the evaluations, providing a structured framework for consistent assessment across all translations. The comparative analysis of GPT-3.5 and GPT-4 is methodologically robust, employing paired t-tests to evaluate differences in performance systematically. This statistical approach minimizes variability and enables precise detection of any meaningful improvements between the AI models. Together, these design elements enhance the validity of the findings and contribute to the study’s relevance in advancing health equity through AI-driven language translation.

The study presented here examines the capabilities of ChatGPT versions 3.5 and 4.0 in translating medical information, with a specific focus on English to Spanish translations of LKD FAQs (47). This study addresses a critical real-world need by tackling barriers faced by Spanish-speaking populations in accessing healthcare information, particularly for LKD. Utilizing actual FAQs from Donate Life America ensures practical relevance, as these FAQs represent common and significant patient inquiries. The consistently high scores in linguistic accuracy (4.89–5.00) and cultural sensitivity (4.89) highlight ChatGPT’s reliability and effectiveness, making a strong case for its integration into healthcare communication strategies. ChatGPT 3.5 achieved an average score of 4.89 in both accuracy and cultural sensitivity. On the other hand, ChatGPT 4.0 marked a significant advancement, attaining a perfect accuracy score of 5.0 while maintaining a similar score in cultural sensitivity as its predecessor.

This study highlights the effectiveness of ChatGPT versions 3.5 and 4.0 in translating predefined FAQs about LKD into Spanish with high levels of accuracy and cultural sensitivity. However, a key advantage of ChatGPT-4 over human translators is its ability to provide instant translations of natural language in real-time interactions. This capability could be particularly impactful in clinical settings where patients frequently ask follow-up questions that require contextually appropriate and accurate responses. For instance, a patient may ask clarifying questions about LKD eligibility, the donation process, or potential risks, to which ChatGPT-4 could provide immediate translations of a physician’s responses, thereby facilitating communication and improving patient understanding. While the current study focused on static translations of FAQs, future research should examine ChatGPT-4’s performance in dynamic, conversational scenarios. This could include simulating patient-physician interactions relevant to LKD topics and assessing ChatGPT-4’s ability to maintain linguistic accuracy, cultural appropriateness, and contextual relevance in real-time exchanges. Such investigations would provide valuable insights into the broader applicability of ChatGPT-4 in enhancing healthcare communication and patient engagement. By expanding its role beyond static translations, ChatGPT-4 could become an invaluable tool in breaking down communication barriers in healthcare settings, particularly for Spanish-speaking patients navigating complex topics like LKD.

These findings not only advance healthcare translation literature but also provide a scalable, impactful solution to bridging language barriers, enhancing health equity, and improving patient education and informed consent in clinical practice. This improvement in accuracy is crucial, indicating the AI’s enhanced ability to accurately translate informational content into another language while preserving the original message’s meaning and intent (56, 57).

The study revealed a key finding. The uniformity in cultural sensitivity scores across both AI versions, despite the notable improvement in linguistic accuracy from ChatGPT 3.5 to 4.0. This uniformity highlights a continuous challenge in AI translation - effectively capturing and conveying cultural meaning. While the linguistic accuracy of the translations showed considerable improvement, the translation of cultural elements did not show similar advancement. This is a significant observation, as it underscores the complex nature of translating not only the language but also the cultural contexts it represents, a crucial aspect in healthcare communication that requires further research and development in AI translation models.

This study adds a unique perspective to the current literature on AI’s role in healthcare communication. Prior research has highlighted the significant impact of language barriers in healthcare settings and suggested technology as a potential solution (6–8, 51, 52). This study extends this discussion by providing empirical evidence of the practical effectiveness of AI, specifically ChatGPT, in medical translations. It demonstrates that advanced AI models are capable of effectively overcoming language barriers, thus enhancing patient understanding and care, and contributing to the reduction of healthcare disparities (58, 59).

This study has notable limitations that warrant acknowledgment and discussion. First, its concentration on a specific set of FAQs from Donate Life America’s website may limit the broader applicability of the findings to other medical areas or languages (47). The number of native Spanish-speaking nephrologist evaluators was not specified, which limits the reproducibility and transparency of the evaluation process. Moreover, the study did not assess inter-rater reliability metrics to quantify agreement among evaluators, potentially introducing variability into the scoring. Additionally, power analysis was not performed to determine whether the sample size of 27 FAQs was sufficient to detect meaningful differences between ChatGPT versions 3.5 and 4.0. Second, the study did not include a comparison of ChatGPT translations with those produced by professional human translators, which would provide valuable context on the relative performance of AI models in medical translation. Furthermore, the evaluation did not account for regional variations in Spanish, which could influence the cultural sensitivity and applicability of the translations to diverse Spanish-speaking populations. While the evaluation was focused on Mexican Spanish, we acknowledge that linguistic and cultural differences exist among Hispanic nations. These distinctions, while not the primary focus of this study, are important considerations for future research aimed at optimizing AI-driven tools like ChatGPT for use in broader Spanish-speaking communities. For this study, the translations were assessed as accessible and culturally appropriate for a general Hispanic audience, though adaptations for regional variations may further enhance their applicability.

The perfect accuracy score for GPT-4 raises the possibility of rating bias or ceiling effects inherent in the evaluation rubric, which could limit the differentiation of performance at the highest levels. These factors emphasize the need for a more nuanced assessment approach that incorporates broader linguistic and cultural perspectives. A key limitation of this study is the absence of participation from Spanish-speaking patients, the primary audience for whom cultural and linguistic appropriateness is most impactful. While evaluations by native Spanish-speaking nephrologists provided expert insights, they may not fully capture the lived experiences and comprehension challenges faced by patients, especially those with limited health literacy. Including a patient sample would have offered direct feedback on usability, cultural relevance, and readability of the translations, which are critical for ensuring effective healthcare communication. Readability, in particular, is vital for populations with low health literacy, and scoring the translations for this aspect would have provided additional insights into their accessibility. Future studies should address this limitation by involving Spanish-speaking patients in the evaluation process and incorporating readability assessments as standard metrics to enhance the practical applicability and impact of AI-generated translations in clinical practice. Future studies should aim to address these limitations by broadening the scope of AI application in translating a wider variety of medical documents across different languages and specifying the number of evaluators. Including inter-rater reliability metrics, conducting power analyses, and comparing AI translations with human translations would further enhance the robustness of future research. Expanding the scope to evaluate regional variations in Spanish and refining evaluation rubrics to mitigate ceiling effects will also improve the generalizability of the findings. By improving the quality and accessibility of translated medical information via AI tools, there is potential to significantly enhance patient outcomes and reduce healthcare disparities, providing a more comprehensive understanding of AI models’ capabilities and their integration into clinical practice for improving health equity.

In conclusion, the study underscores the potential of AI language models, particularly ChatGPT versions 3.5 and 4.0, in providing accurate and culturally sensitive translations of medical information. These findings highlight the significant role that AI can play in overcoming language barriers in healthcare, contributing to improved patient education and, ultimately, to the reduction of healthcare access disparities. As AI technology continues to progress and evolve, its integration into various medical contexts is likely to become more widespread, further supporting the objective of achieving health equity and improving the quality of patient care. However, continuous research and development efforts are necessary to address the challenges in translating cultural complexities and to ensure the generalizability of AI-based translation solutions across diverse medical contexts and languages.

## Data Availability

The original contributions presented in the study are included in the article/Supplementary material, further inquiries can be directed to the corresponding author.
