# Supplementary material for: Advancing health equity: evaluating AI translations of kidney donor information for Spanish speakers
Source: Front Public Health. 2025 Jan 27;13:1484790. doi: 10.3389/fpubh.2025.1484790 (PMC11808013; doi:10.3389/fpubh.2025.1484790)
Supplement: Supplementary file 1 [file Data_Sheet_1.docx]

**Table S1. The score for linguistic accuracy and cultural sensitivity of GPT-3.5 and GPT-4.0 for individual FAQs**

| **Chat GPT 3.5** | | | **Chat GPT 4.0** | | |
| --- | --- | --- | --- | --- | --- |
| Question number | Accuracy | Culture | Question number | Accuracy | Culture |
| 1 | 4 | 5 | 1 | 5 | 5 |
| 2 | 5 | 5 | 2 | 5 | 5 |
| 3 | 5 | 5 | 3 | 5 | 5 |
| 4 | 5 | 5 | 4 | 5 | 5 |
| 5 | 5 | 5 | 5 | 5 | 4 |
| 6 | 5 | 5 | 6 | 5 | 5 |
| 7 | 5 | 5 | 7 | 5 | 5 |
| 8 | 5 | 5 | 8 | 5 | 5 |
| 9 | 5 | 5 | 9 | 5 | 5 |
| 10 | 5 | 5 | 10 | 5 | 5 |
| 11 | 5 | 5 | 11 | 5 | 5 |
| 12 | 5 | 5 | 12 | 5 | 5 |
| 13 | 5 | 5 | 13 | 5 | 5 |
| 14 | 5 | 5 | 14 | 5 | 5 |
| 15 | 5 | 4 | 15 | 5 | 5 |
| 16 | 4 | 5 | 16 | 5 | 5 |
| 17 | 5 | 5 | 17 | 5 | 5 |
| 18 | 4 | 5 | 18 | 5 | 5 |
| 19 | 5 | 5 | 19 | 5 | 5 |
| 20 | 5 | 5 | 20 | 5 | 5 |
| 21 | 5 | 5 | 21 | 5 | 5 |
| 22 | 5 | 5 | 22 | 5 | 5 |
| 23 | 5 | 5 | 23 | 5 | 5 |
| 24 | 5 | 5 | 24 | 5 | 5 |
| 25 | 5 | 5 | 25 | 5 | 5 |
| 26 | 5 | 4 | 26 | 5 | 4 |
| 27 | 5 | 4 | 27 | 5 | 4 |
| Average | 4.89 | 4.89 | Average | 5.00 | 4.89 |

Chat GPT 3.5

1. Please translate to Spanish: "What is living donation? Living donation is when a living person donates an organ or part of an organ to a person in need of a transplant. Living kidney donation is possible because we can live a healthy life with one functioning kidney. Living liver donation is possible because the liver consists of two lobes, one of which can be donated to someone in need. Both lobes will regenerate to normal size and function generally within 6-12 weeks."

ChatGPT

**ChatGPT**

¿Qué es la donación en vida? La donación en vida es cuando una persona viva dona un órgano o parte de un órgano a una persona que necesita un trasplante. La donación de riñón en vida es posible porque podemos llevar una vida saludable con un riñón funcionante. La donación de hígado en vida es posible porque el hígado consta de dos lóbulos, uno de los cuales puede donarse a alguien necesitado. Ambos lóbulos se regenerarán hasta su tamaño y función normales generalmente en un plazo de 6 a 12 semanas.


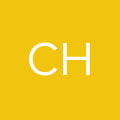


**You**

1. Please translate to Spanish: Why is living donation important? Living donation offers another choice for transplant candidates, and reduces the number of people on the national transplant waiting list (from deceased donors). Even better, kidney and liver patients who are able to receive a living donor transplant can receive a good quality organ much sooner, often in less than a year. The limited number of deceased donor organs cannot meet the growing need for lifesaving organ transplants. Living donation is one way we can give more people access to the lifesaving transplant they need. Other benefits of living donation include: The recipient can be transplanted while in better health and better able to tolerate the surgery. Living donor organs are often better quality and on average last longer than deceased donor organs. Kidney disease patients can avoid years of dialysis. The surgery can be scheduled at a time convenient for both the donor and the recipient. Many living donors refer to the experience as one of their most rewarding achievements."

ChatGPT

**ChatGPT**

¿Por qué es importante la donación en vida? La donación en vida ofrece otra opción para los candidatos a trasplantes y reduce el número de personas en la lista nacional de espera de trasplantes (de donantes fallecidos). Incluso mejor, los pacientes de riñón e hígado que pueden recibir un trasplante de un donante vivo pueden obtener un órgano de buena calidad mucho más rápido, a menudo en menos de un año. El número limitado de órganos de donantes fallecidos no puede satisfacer la creciente necesidad de trasplantes de órganos que salvan vidas. La donación en vida es una forma de brindar a más personas acceso al trasplante que necesitan para salvar sus vidas. Otros beneficios de la donación en vida incluyen: El receptor puede ser trasplantado estando en mejor salud y mejor preparado para tolerar la cirugía. Los órganos de donantes vivos suelen tener mejor calidad y, en promedio, duran más que los órganos de donantes fallecidos. Los pacientes con enfermedad renal pueden evitar años de diálisis. La cirugía se puede programar en un momento conveniente tanto para el donante como para el receptor. Muchos donantes vivos consideran la experiencia como uno de sus logros más gratificantes."


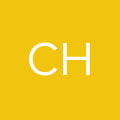


**You**

1. Please translate to Spanish: "Does the living donor need to know the person to whom they donate an organ? No. A person may donate to a specific person they know or anonymously to someone in need. This would be a non-directed donation."

ChatGPT

**ChatGPT**

"¿El donante en vida necesita conocer a la persona a quien dona un órgano? No. Una persona puede donar a una persona específica que conoce o de manera anónima a alguien que lo necesite. Esto se consideraría una donación no dirigida."


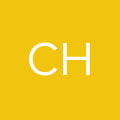


**You**

1. Please translate to Spanish: "What is involved in the evaluation to be a living donor? The evaluation is designed to protect both the donor and the recipient. It ensures that the donor is healthy enough for the surgery and is making an informed decision. A potential living donor undergoes both physical and psychosocial examinations. Testing can vary depending on the organ and donor’s age. In addition, routine health screens will need to be up to date before donation can occur. Learn more about living donation health screens from the American Society of Transplantation (AST)."

ChatGPT

**ChatGPT**

"¿Qué implica la evaluación para ser un donante en vida? La evaluación está diseñada para proteger tanto al donante como al receptor. Asegura que el donante esté lo suficientemente saludable para la cirugía y esté tomando una decisión informada. Un posible donante en vida se somete a exámenes físicos y psicosociales. Las pruebas pueden variar según el órgano y la edad del donante. Además, los controles de salud de rutina deberán estar actualizados antes de que pueda ocurrir la donación. Obtenga más información sobre los controles de salud para la donación en vida en la Sociedad Americana de Trasplantes (AST, por sus siglas en inglés)."


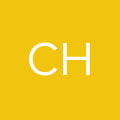


**You**

1. Please translate to Spanish: "What is paired kidney donation and living donation chains? Sometimes a transplant candidate has someone who wants to donate a kidney to them, but tests reveal that the kidney would not be a good medical match. Paired donation, also called paired kidney exchange, gives that transplant candidate another option. In paired donation, two or more pairs of living kidney donors and the individuals they want to donate to are swapped to form a compatible medical match. For more detailed information, check with your transplant hospital or learn more about paired kidney donation from UNOS.org"

ChatGPT

**ChatGPT**

"¿Qué es la donación de riñón emparejada y las cadenas de donación en vida? A veces, un candidato a trasplante tiene a alguien que quiere donarle un riñón, pero las pruebas revelan que el riñón no sería una buena coincidencia médica. La donación emparejada, también llamada intercambio de riñones emparejados, ofrece a ese candidato a trasplante otra opción. En la donación emparejada, dos o más pares de donantes vivos de riñón y las personas a las que desean donarles se intercambian para formar una coincidencia médica compatible. Para obtener información más detallada, consulte con su hospital de trasplantes o obtenga más información sobre la donación de riñón emparejada en UNOS.org."


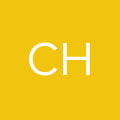


**You**

1. Please translate to Spanish: "Can living kidney donors live a healthy life with a single kidney? Studies suggest that people can live a normal, healthy life with one kidney. Living kidney donors are carefully screened to ensure they are healthy before living donation can take place. They are encouraged to have regular checkups and follow a healthy lifestyle."

ChatGPT

**ChatGPT**

"¿Pueden los donantes de riñón en vida llevar una vida saludable con un solo riñón? Estudios sugieren que las personas pueden llevar una vida normal y saludable con un solo riñón. Los donantes de riñón en vida son sometidos a un exhaustivo proceso de evaluación para garantizar que estén sanos antes de que se lleve a cabo la donación en vida. Se les anima a realizarse chequeos regulares y a llevar un estilo de vida saludable."


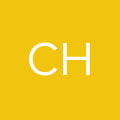


**You**

1. Please translate to Spanish: "Does living organ donation shorten the donor’s life expectancy? No, there is no evidence that living donation shortens a lifespan."

ChatGPT

**ChatGPT**

"¿La donación de órganos en vida acorta la esperanza de vida del donante? No, no existe evidencia de que la donación en vida acorte la esperanza de vida."


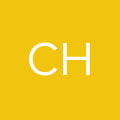


**You**

1. Please translate to Spanish: "Are living kidney donors more likely to get kidney disease? Living kidney donors are counseled about their individual risks associated with donation. The transplant team educates donors about the expected post-donation kidney function. In general, the risk of kidney disease does not exceed that of the general population with the same demographic profile"

ChatGPT

**ChatGPT**

"¿Los donantes de riñón en vida tienen más probabilidades de contraer enfermedad renal? Los donantes de riñón en vida reciben asesoramiento sobre los riesgos individuales asociados con la donación. El equipo de trasplantes educa a los donantes sobre la función esperada del riñón después de la donación. En general, el riesgo de enfermedad renal no supera el de la población general con el mismo perfil demográfico."


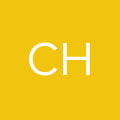


**You**

1. Please translate to Spanish: "Do living donors have to take medications for the rest of their lives? Generally, prescriptions for pain and stool softeners will be necessary in the immediate post-operative period only."

ChatGPT

**ChatGPT**

"¿Los donantes en vida tienen que tomar medicamentos por el resto de sus vidas? Por lo general, las recetas de analgésicos y ablandadores de heces solo serán necesarias en el período inmediato posterior a la cirugía."


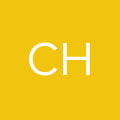


**You**

1. Please translate to Spanish: "Will a living donor be in the hospital for an extended period of time after surgery? A kidney donor will be hospitalized on average for 1 to 2 nights. A liver donor will be hospitalized on average for 4 to 7 nights. Liver donors may spend at least 1 night in the ICU for monitoring. A donor will have some follow-up appointments after hospital discharge."

ChatGPT

**ChatGPT**

"¿Estará un donante en vida en el hospital durante un período prolongado después de la cirugía? Un donante de riñón estará hospitalizado en promedio de 1 a 2 noches. Un donante de hígado estará hospitalizado en promedio de 4 a 7 noches. Los donantes de hígado pueden pasar al menos 1 noche en la UCI para su monitoreo. Un donante tendrá algunas citas de seguimiento después del alta hospitalaria."


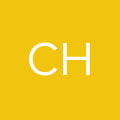


**You**

1. Please translate to Spanish: "Does a living donor have to follow a new diet plan following donation? A donor should eat a healthy, well-balanced diet, but there are no dietary restrictions."

ChatGPT

**ChatGPT**

"¿Debe un donante en vida seguir un nuevo plan de dieta después de la donación? Un donante debe seguir una dieta saludable y equilibrada, pero no hay restricciones dietéticas."


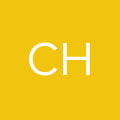


**You**

1. Please translate to Spanish: "Can a living donor consume alcohol following donation? Excessive alcohol use is always dangerous. For liver donors, alcohol abstinence should occur for one month prior to surgery and for three months following donation. No specific restrictions for kidney donors."

ChatGPT

**ChatGPT**

"¿Puede un donante en vida consumir alcohol después de la donación? El consumo excesivo de alcohol siempre es peligroso. Para los donantes de hígado, la abstinencia de alcohol debe ocurrir durante un mes antes de la cirugía y durante tres meses después de la donación. No hay restricciones específicas para los donantes de riñón."


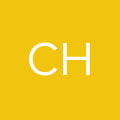


**You**

1. Please translate to Spanish: "Should a living donor avoid pregnancy after donation? A donor should wait to become pregnant until medically cleared after donation."

ChatGPT

**ChatGPT**

"¿Debería un donante en vida evitar el embarazo después de la donación? Un donante debe esperar para quedar embarazada hasta que se le haya dado el visto bueno médico después de la donación."


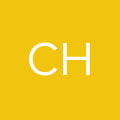


**You**

1. Please translate to Spanish: "Will a living donor’s sex life be negatively affected by donation? Donors may engage in sexual activity when they feel well enough to do so."

ChatGPT

**ChatGPT**

"¿La vida sexual de un donante en vida se verá afectada negativamente por la donación? Los donantes pueden participar en actividad sexual cuando se sientan lo suficientemente bien para hacerlo."


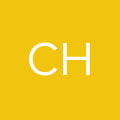


**You**

1. Please translate to Spanish: "Who pays for the medical expenses related to living donation? The costs for the medical testing, surgery and post-operative care are covered by the transplant hospital or recipient’s health insurance. However, the living donor is responsible for the cost of routine health screens and treatment of any medical issues discovered during the evaluation. The cost of treatment of future issues that result from the donation, may be covered. Medical costs that are not covered include post-surgical routine health maintenance. Employed living donors should learn about their company’s paid sick leave, disability and the Family and Medical Leave Act (FMLA) policies if applicable. A transplant financial coordinator is available at each transplant hospital to answer questions."

ChatGPT

**ChatGPT**

"¿Quién paga los gastos médicos relacionados con la donación en vida? Los costos de las pruebas médicas, la cirugía y la atención postoperatoria están cubiertos por el hospital de trasplantes o el seguro de salud del receptor. Sin embargo, el donante en vida es responsable de los costos de las pruebas de salud de rutina y del tratamiento de cualquier problema médico descubierto durante la evaluación. Los costos de tratamiento de problemas futuros que resulten de la donación pueden estar cubiertos. Los costos médicos que no están cubiertos incluyen el mantenimiento de rutina de la salud después de la cirugía. Los donantes en vida empleados deben informarse sobre las políticas de licencia por enfermedad remunerada de su empresa, la discapacidad y la Ley de Licencia Familiar y Médica (FMLA) si corresponde. En cada hospital de trasplantes, hay un coordinador financiero de trasplantes disponible para responder a preguntas."


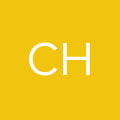


**You**

1. Please translate to Spanish: "Will the living donor have any out of pocket expenses? Yes. It varies case by case and could include lost wages, travel expenses, childcare costs, etc. The National Organ Transplant Act allows for organ recipients to pay their living donors’ travel and housing in connection with the donation. The transplant hospital financial coordinator will check with the insurance company of the intended recipient as the policy may provide a travel benefit. There are organizations that provide financial resources to living donors for uncovered costs depending on need. Transplant hospitals can provide additional information about options. National Living Donor Assistance Center (NLDAC) Note that eligibility for financial assistance from NLDAC is based on the recipient’s household income which may not be more than 300% of the current Federal Poverty Guidelines."

ChatGPT

**ChatGPT**

"¿Tendrá el donante en vida algún gasto de su bolsillo? Sí, varía de caso en caso y puede incluir salarios perdidos, gastos de viaje, costos de cuidado de niños, entre otros. La Ley Nacional de Trasplantes de Órganos permite que los receptores de órganos paguen los gastos de viaje y alojamiento de sus donantes en vida en relación con la donación. El coordinador financiero del hospital de trasplantes verificará con la compañía de seguros del receptor previsto, ya que la póliza puede proporcionar un beneficio de viaje. Existente organizaciones que proporcionan recursos financieros a donantes en vida para cubrir costos no incluidos, según la necesidad. Los hospitales de trasplantes pueden proporcionar información adicional sobre las opciones. Centro Nacional de Asistencia para Donantes en Vida (NLDAC, por sus siglas en inglés). Tenga en cuenta que la elegibilidad para recibir asistencia financiera del NLDAC se basa en los ingresos del hogar del receptor, que no pueden superar el 300% de las Pautas Federales de Pobreza vigentes."


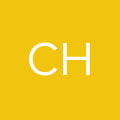


**You**

1. Please translate to Spanish: "Will a living donor have trouble getting health insurance or life insurance after they donate? Being a living donor may affect the ability to get health or life insurance. The transplant team can provide details."

ChatGPT

**ChatGPT**

"¿Tendrá un donante en vida problemas para obtener un seguro de salud o un seguro de vida después de la donación? Ser un donante en vida puede afectar la capacidad para obtener un seguro de salud o de vida. El equipo de trasplantes puede proporcionar detalles al respecto."


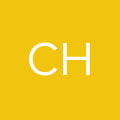


**You**

1. Please translate to Spanish: "How risky is living donor surgery? The surgery to donate a kidney is generally safe, with a 3 in 10,000 risk of death. The risks for living liver donation surgery are slightly higher, dependent on the amount of liver donated. More information about living donation risks is available on the United Network for Organ Sharing (UNOS) website, TransplantLiving.org. As with any surgery, living-liver donation can include complications such as bleeding, infection, bile duct problems, and blood clots, etc. Complications occur in about 16% of cases. Living donors are carefully evaluated prior to a living-donor liver transplant to ensure that risk is minimized. The estimated mortality is about 0.5% (5/1000), depending on which lobe (part) of the liver is donated."

ChatGPT

**ChatGPT**

"¿Qué tan arriesgada es la cirugía para donantes en vida? La cirugía para donar un riñón generalmente es segura, con un riesgo de muerte de 3 de cada 10,000 casos. Los riesgos para la cirugía de donación de hígado en vida son ligeramente más altos, dependiendo de la cantidad de hígado donado. Puede encontrar más información sobre los riesgos de la donación en vida en el sitio web de la United Network for Organ Sharing (UNOS), TransplantLiving.org. Al igual que con cualquier cirugía, la donación de hígado en vida puede incluir complicaciones como sangrado, infección, problemas en los conductos biliares y coágulos de sangre, entre otros. Las complicaciones ocurren en aproximadamente el 16% de los casos. Los donantes en vida son cuidadosamente evaluados antes de un trasplante de hígado de donante en vida para minimizar los riesgos. La mortalidad estimada es de aproximadamente 0.5% (5/1000), dependiendo de qué lóbulo (parte) del hígado se done."


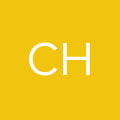


**You**

1. Please translate to Spanish: "Can adults over age 50 donate? Yes, donors must be healthy and can donate primarily on a basis of health. Some transplant hospitals may have age restrictions."

ChatGPT

**ChatGPT**

"¿Pueden los adultos mayores de 50 años donar? Sí, los donantes deben estar sanos y pueden donar principalmente en función de su salud. Algunos hospitales de trasplantes pueden tener restricciones de edad."


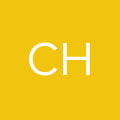


**You**

1. Please translate to Spanish: "Can members of the LGBTQ+ community be living donors? There are no restrictions based on a potential donor’s LGBTQ+ identification. All potential living donors undergo a thorough medical evaluation to determine if donation is safe."

ChatGPT

**ChatGPT**

"¿Pueden los miembros de la comunidad LGBTQ+ ser donantes en vida? No hay restricciones basadas en la identificación LGBTQ+ de un posible donante. Todos los posibles donantes en vida se someten a una exhaustiva evaluación médica para determinar si la donación es segura."


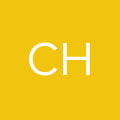


**You**

1. Please translate to Spanish: "Can people living with HIV be living donors? The HIV Organ Policy Equity Act (HOPE) Act provides donation and transplantation opportunities for people living with HIV with minimal additional testing at specific centers. Refer to the OPTN HOPE (HIV Organ Policy Equity) Act page for more information."

ChatGPT

**ChatGPT**

"¿Pueden las personas que viven con el VIH ser donantes en vida? La Ley de Equidad de Política de Órganos del VIH (HOPE) ofrece oportunidades de donación y trasplante para personas que viven con el VIH con pruebas adicionales mínimas en centros específicos. Consulte la página de la OPTN HOPE (Ley de Equidad de Política de Órganos del VIH) para obtener más información."


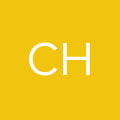


**You**

1. Please translate to Spanish: "Are people with tattoos able to be living donors? All potential living donors undergo a thorough medical evaluation to determine if donation is safe for both the donor and recipient."

ChatGPT

**ChatGPT**

"¿Pueden las personas con tatuajes ser donantes en vida? Todos los posibles donantes en vida se someten a una exhaustiva evaluación médica para determinar si la donación es segura tanto para el donante como para el receptor."


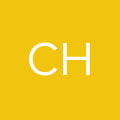


**You**

1. Please translate to Spanish: "What happens if a living donor changes their mind? Being a living donor is completely voluntary. The donor can delay or stop the process at any time. Their reasons for choosing to not donate will remain confidential and will not be shared with the recipient or anyone else."

ChatGPT

**ChatGPT**

"¿Qué sucede si un donante en vida cambia de opinión? Ser un donante en vida es completamente voluntario. El donante puede retrasar o detener el proceso en cualquier momento. Sus razones para no querer donar se mantendrán confidenciales y no se compartirán con el receptor ni con nadie más."


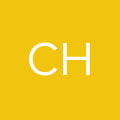


**You**

1. Please translate to Spanish: "Do living donors ever share their experiences and what are some resources? Yes, 97% of living donors say they would donate again and are excited to share their experiences. Donate Life WELD (We Encourage Living Donation), a program of Donate Life America, provides a forum for living donors to share their stories with members of their community. To learn more about DL/WELD, contact DL/WELD coordinator. View stories from living donors and recipients."

ChatGPT

**ChatGPT**

"¿Comparten alguna vez los donantes en vida sus experiencias y cuáles son algunos recursos? Sí, el 97% de los donantes en vida dicen que donarían de nuevo y están emocionados de compartir sus experiencias. Donate Life WELD (We Encourage Living Donation), un programa de Donate Life America, proporciona un espacio para que los donantes en vida compartan sus historias con miembros de su comunidad. Para obtener más información sobre DL/WELD, contacte al coordinador de DL/WELD. Vea las historias de donantes en vida y receptores."


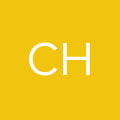


**You**

1. Please translate to Spanish: "How would a potential living donor start the process if they want to donate to a specific person? Contacting the transplant hospital of the person in need of the transplant is the first step. Many hospitals have a form on their website potential donors can submit to begin the process or they can call the transplant hospital and ask to speak with the living donor coordinator for the organ they wish to donate."

ChatGPT

**ChatGPT**

"¿Cómo comenzaría un posible donante en vida el proceso si desea donar a una persona específica? El primer paso es ponerse en contacto con el hospital de trasplantes de la persona que necesita el trasplante. Muchos hospitales tienen un formulario en su sitio web que los posibles donantes pueden enviar para comenzar el proceso, o pueden llamar al hospital de trasplantes y solicitar hablar con el coordinador de donantes en vida del órgano que desean donar."


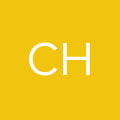


**You**

1. Please translate to Spanish: "What if the intended living kidney donor and intended recipient are not a match? Begin by talking to the transplant hospital where the intended recipient is listed to see if they participate in paired donation. The OPTN Kidney Paired Donation Program facilitates paired donation between transplant hospitals."

ChatGPT

**ChatGPT**

"¿Qué sucede si el donante de riñón en vida previsto y el receptor previsto no son compatibles? Comience hablando con el hospital de trasplantes donde está registrado el receptor previsto para ver si participan en la donación emparejada. El Programa de Donación de Riñón en Parejas de OPTN facilita la donación emparejada entre hospitales de trasplantes."


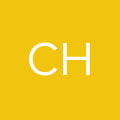


**You**

1. Please translate to Spanish: "What is the first step for a potential living donor who does not have a specific recipient in mind? Please refer to the Organ Procurement and Transplantation Network (OPTN) member list of all U.S. transplant hospitals. You may also want to consider being part of a living kidney donor paired exchange or chain. (see above)"

ChatGPT

**ChatGPT**

"¿Cuál es el primer paso para un posible donante en vida que no tiene un receptor específico en mente? Por favor, consulte la lista de miembros de la Red de Procuración y Trasplante de Órganos (OPTN) de todos los hospitales de trasplantes de EE. UU. También puede considerar ser parte de un intercambio emparejado de donantes de riñón en vida o una cadena de donantes (ver arriba)."

Chat GPT 4.0

**You**

1. Please translate to Spanish: "What is living donation? Living donation is when a living person donates an organ or part of an organ to a person in need of a transplant. Living kidney donation is possible because we can live a healthy life with one functioning kidney. Living liver donation is possible because the liver consists of two lobes, one of which can be donated to someone in need. Both lobes will regenerate to normal size and function generally within 6-12 weeks."

ChatGPT

**ChatGPT**

Sure, here is the translation to Spanish:

"¿Qué es la donación en vida? La donación en vida ocurre cuando una persona viva dona un órgano o parte de un órgano a alguien que necesita un trasplante. La donación de riñón en vida es posible porque podemos vivir una vida saludable con un solo riñón funcionando. La donación de hígado en vida es posible porque el hígado consta de dos lóbulos, uno de los cuales puede ser donado a alguien en necesidad. Ambos lóbulos se regenerarán hasta alcanzar un tamaño y función normales generalmente dentro de 6 a 12 semanas."


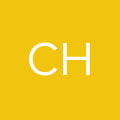


**You**

1. Please translate to Spanish: Why is living donation important? Living donation offers another choice for transplant candidates, and reduces the number of people on the national transplant waiting list (from deceased donors). Even better, kidney and liver patients who are able to receive a living donor transplant can receive a good quality organ much sooner, often in less than a year. The limited number of deceased donor organs cannot meet the growing need for lifesaving organ transplants. Living donation is one way we can give more people access to the lifesaving transplant they need. Other benefits of living donation include: The recipient can be transplanted while in better health and better able to tolerate the surgery. Living donor organs are often better quality and on average last longer than deceased donor organs. Kidney disease patients can avoid years of dialysis. The surgery can be scheduled at a time convenient for both the donor and the recipient. Many living donors refer to the experience as one of their most rewarding achievements."

2 / 2

ChatGPT

**ChatGPT**

Certainly, here's the translation to Spanish:

"¿Por qué es importante la donación en vida? La donación en vida ofrece otra opción para los candidatos a trasplante y reduce el número de personas en la lista de espera nacional de trasplantes (de donantes fallecidos). Incluso mejor, los pacientes de riñón e hígado que pueden recibir un trasplante de un donante vivo pueden recibir un órgano de buena calidad mucho más pronto, a menudo en menos de un año.

El número limitado de órganos de donantes fallecidos no puede satisfacer la creciente necesidad de trasplantes de órganos vitales. La donación en vida es una manera en que podemos dar a más personas acceso al trasplante vital que necesitan. Otros beneficios de la donación en vida incluyen:

El receptor puede ser trasplantado mientras se encuentra en mejor estado de salud y más capacitado para tolerar la cirugía. Los órganos de donantes vivos suelen ser de mejor calidad y en promedio duran más que los órganos de donantes fallecidos. Los pacientes con enfermedad renal pueden evitar años de diálisis. La cirugía puede programarse en un momento conveniente tanto para el donante como para el receptor. Muchos donantes vivos describen la experiencia como uno de sus logros más gratificantes."


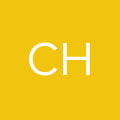


**You**

1. Please translate to Spanish: "Does the living donor need to know the person to whom they donate an organ? No. A person may donate to a specific person they know or anonymously to someone in need. This would be a non-directed donation."

ChatGPT

**ChatGPT**

Sure, here's the translation to Spanish:

"¿Necesita el donante vivo conocer a la persona a la que dona un órgano? No. Una persona puede donar a alguien específico que conozca o de manera anónima a alguien en necesidad. Esto sería una donación no dirigida."


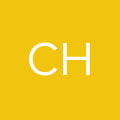


**You**

1. Please translate to Spanish: "What is involved in the evaluation to be a living donor? The evaluation is designed to protect both the donor and the recipient. It ensures that the donor is healthy enough for the surgery and is making an informed decision. A potential living donor undergoes both physical and psychosocial examinations. Testing can vary depending on the organ and donor’s age. In addition, routine health screens will need to be up to date before donation can occur. Learn more about living donation health screens from the American Society of Transplantation (AST)."

ChatGPT

**ChatGPT**

Certainly, here is the Spanish translation:

"¿Qué implica la evaluación para ser un donante vivo? La evaluación está diseñada para proteger tanto al donante como al receptor. Asegura que el donante esté lo suficientemente saludable para la cirugía y que esté tomando una decisión informada. Un posible donante vivo se somete tanto a exámenes físicos como psicosociales. Las pruebas pueden variar dependiendo del órgano y de la edad del donante. Además, las pruebas de salud rutinarias deberán estar actualizadas antes de que pueda realizarse la donación. Obtenga más información sobre las pruebas de salud para la donación en vida de la Sociedad Americana de Trasplantes (AST)."


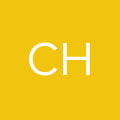


**You**

1. Please translate to Spanish: "What is paired kidney donation and living donation chains? Sometimes a transplant candidate has someone who wants to donate a kidney to them, but tests reveal that the kidney would not be a good medical match. Paired donation, also called paired kidney exchange, gives that transplant candidate another option. In paired donation, two or more pairs of living kidney donors and the individuals they want to donate to are swapped to form a compatible medical match. For more detailed information, check with your transplant hospital or learn more about paired kidney donation from UNOS.org"

ChatGPT

**ChatGPT**

Sure, here's the translation to Spanish:

"¿Qué son la donación de riñón apareada y las cadenas de donación en vida? A veces, un candidato a trasplante tiene a alguien que quiere donarle un riñón, pero las pruebas revelan que el riñón no sería una buena compatibilidad médica. La donación apareada, también conocida como intercambio de riñón apareado, ofrece a ese candidato a trasplante otra opción. En la donación apareada, dos o más pares de donantes de riñón vivos y las personas a las que quieren donar se intercambian para formar una compatibilidad médica adecuada. Para obtener información más detallada, consulte con su hospital de trasplante o aprenda más sobre la donación de riñón apareada en UNOS.org"


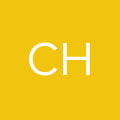


**You**

1. Please translate to Spanish: "Can living kidney donors live a healthy life with a single kidney? Studies suggest that people can live a normal, healthy life with one kidney. Living kidney donors are carefully screened to ensure they are healthy before living donation can take place. They are encouraged to have regular checkups and follow a healthy lifestyle."

ChatGPT

**ChatGPT**

Certainly, here's the translation to Spanish:

"¿Pueden los donantes de riñón en vida vivir una vida saludable con un solo riñón? Estudios sugieren que las personas pueden vivir una vida normal y saludable con un solo riñón. Los donantes de riñón en vida son cuidadosamente evaluados para asegurarse de que estén saludables antes de que pueda realizarse la donación en vida. Se les anima a tener chequeos regulares y seguir un estilo de vida saludable."


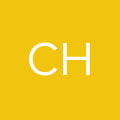


**You**

1. Please translate to Spanish: "Does living organ donation shorten the donor’s life expectancy? No, there is no evidence that living donation shortens a lifespan."

ChatGPT

**ChatGPT**

Sure, here's the translation to Spanish:

"¿Acorta la donación de órganos en vida la esperanza de vida del donante? No, no hay evidencia de que la donación en vida acorte la esperanza de vida."


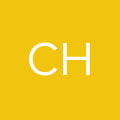


**You**

1. Please translate to Spanish: "Are living kidney donors more likely to get kidney disease? Living kidney donors are counseled about their individual risks associated with donation. The transplant team educates donors about the expected post-donation kidney function. In general, the risk of kidney disease does not exceed that of the general population with the same demographic profile"

ChatGPT

**ChatGPT**

Certainly, here's the Spanish translation:

"¿Es más probable que los donantes de riñón en vida desarrollen enfermedades renales? Los donantes de riñón en vida son informados sobre sus riesgos individuales asociados con la donación. El equipo de trasplante educa a los donantes sobre la función renal esperada después de la donación. En general, el riesgo de enfermedad renal no excede al de la población general con el mismo perfil demográfico."


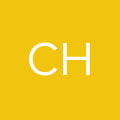


**You**

1. Please translate to Spanish: "Do living donors have to take medications for the rest of their lives? Generally, prescriptions for pain and stool softeners will be necessary in the immediate post-operative period only."

ChatGPT

**ChatGPT**

Sure, here's the translation to Spanish:

"¿Tienen que tomar medicamentos los donantes vivos por el resto de sus vidas? Generalmente, se necesitarán recetas para el dolor y ablandadores de heces solo en el período inmediato posterior a la operación."


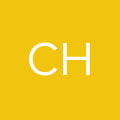


**You**

1. Please translate to Spanish: "Will a living donor be in the hospital for an extended period of time after surgery? A kidney donor will be hospitalized on average for 1 to 2 nights. A liver donor will be hospitalized on average for 4 to 7 nights. Liver donors may spend at least 1 night in the ICU for monitoring. A donor will have some follow-up appointments after hospital discharge."

ChatGPT

**ChatGPT**

Sure, here's the translation to Spanish:

"¿Estará un donante vivo en el hospital por un período prolongado de tiempo después de la cirugía? Un donante de riñón estará hospitalizado en promedio de 1 a 2 noches. Un donante de hígado estará hospitalizado en promedio de 4 a 7 noches. Los donantes de hígado pueden pasar al menos 1 noche en la UCI para monitoreo. Un donante tendrá algunas citas de seguimiento después del alta hospitalaria."


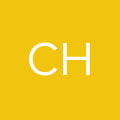


**You**

1. Please translate to Spanish: "Does a living donor have to follow a new diet plan following donation? A donor should eat a healthy, well-balanced diet, but there are no dietary restrictions."

2 / 2

ChatGPT

**ChatGPT**

Certainly, here's the translation to Spanish:

"¿Tiene que seguir un donante vivo un nuevo plan de dieta después de la donación? Un donante debe comer una dieta saludable y equilibrada, pero no hay restricciones dietéticas."


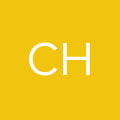


**You**

1. Please translate to Spanish: "Can a living donor consume alcohol following donation? Excessive alcohol use is always dangerous. For liver donors, alcohol abstinence should occur for one month prior to surgery and for three months following donation. No specific restrictions for kidney donors."

ChatGPT

**ChatGPT**

Certainly, here's the Spanish translation:

"¿Puede un donante vivo consumir alcohol después de la donación? El consumo excesivo de alcohol es siempre peligroso. Para los donantes de hígado, se debe abstener del alcohol durante un mes antes de la cirugía y durante tres meses después de la donación. No hay restricciones específicas para los donantes de riñón."


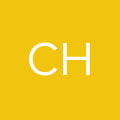


**You**

1. Please translate to Spanish: "Should a living donor avoid pregnancy after donation? A donor should wait to become pregnant until medically cleared after donation."

ChatGPT

**ChatGPT**

Sure, here's the translation to Spanish:

"¿Debería un donante vivo evitar el embarazo después de la donación? Un donante debe esperar para quedar embarazada hasta que sea médicamente autorizada después de la donación."


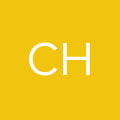


**You**

1. Please translate to Spanish: "Will a living donor’s sex life be negatively affected by donation? Donors may engage in sexual activity when they feel well enough to do so."

ChatGPT

**ChatGPT**

Certainly, here's the translation to Spanish:

"¿Se verá afectada negativamente la vida sexual de un donante vivo por la donación? Los donantes pueden participar en actividad sexual cuando se sientan lo suficientemente bien como para hacerlo."


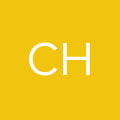


**You**

1. Please translate to Spanish: "Who pays for the medical expenses related to living donation? The costs for the medical testing, surgery and post-operative care are covered by the transplant hospital or recipient’s health insurance. However, the living donor is responsible for the cost of routine health screens and treatment of any medical issues discovered during the evaluation. The cost of treatment of future issues that result from the donation, may be covered. Medical costs that are not covered include post-surgical routine health maintenance. Employed living donors should learn about their company’s paid sick leave, disability and the Family and Medical Leave Act (FMLA) policies if applicable. A transplant financial coordinator is available at each transplant hospital to answer questions."

ChatGPT

**ChatGPT**

Sure, here's the translation to Spanish:

"¿Quién paga los gastos médicos relacionados con la donación en vida? Los costos de las pruebas médicas, la cirugía y el cuidado postoperatorio son cubiertos por el hospital de trasplante o el seguro de salud del receptor. Sin embargo, el donante vivo es responsable del costo de las pruebas de salud rutinarias y del tratamiento de cualquier problema médico descubierto durante la evaluación. El costo del tratamiento de problemas futuros que resulten de la donación, puede ser cubierto. Los costos médicos que no están cubiertos incluyen el mantenimiento de la salud rutinaria postquirúrgica. Los donantes vivos empleados deben informarse sobre las políticas de su compañía en cuanto a la licencia por enfermedad pagada, discapacidad y la Ley de Ausencia Familiar y Médica (FMLA) si corresponde. Un coordinador financiero de trasplantes está disponible en cada hospital de trasplante para responder preguntas."

1 / 2


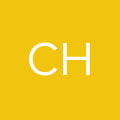


**You**

1. Please translate to Spanish: "Will the living donor have any out of pocket expenses? Yes. It varies case by case and could include lost wages, travel expenses, childcare costs, etc. The National Organ Transplant Act allows for organ recipients to pay their living donors’ travel and housing in connection with the donation. The transplant hospital financial coordinator will check with the insurance company of the intended recipient as the policy may provide a travel benefit. There are organizations that provide financial resources to living donors for uncovered costs depending on need. Transplant hospitals can provide additional information about options. National Living Donor Assistance Center (NLDAC) Note that eligibility for financial assistance from NLDAC is based on the recipient’s household income which may not be more than 300% of the current Federal Poverty Guidelines."

ChatGPT

**ChatGPT**

Certainly, here's the translation to Spanish:

"¿Tendrá el donante vivo algún gasto de bolsillo? Sí. Varía de caso a caso e incluye posibles gastos como la pérdida de salarios, gastos de viaje, costos de cuidado infantil, etc. La Ley Nacional de Trasplante de Órganos permite a los receptores de órganos pagar los gastos de viaje y alojamiento de sus donantes vivos en relación con la donación. El coordinador financiero del hospital de trasplante verificará con la compañía de seguros del receptor previsto, ya que la póliza puede ofrecer un beneficio de viaje. Hay organizaciones que brindan recursos financieros a los donantes vivos para cubrir los costos no cubiertos dependiendo de la necesidad. Los hospitales de trasplante pueden proporcionar información adicional sobre las opciones.

Centro Nacional de Asistencia para Donantes Vivos (NLDAC) Tenga en cuenta que la elegibilidad para la asistencia financiera de NLDAC se basa en el ingreso familiar del receptor, que no puede ser más del 300% de las Pautas Federales de Pobreza actuales."


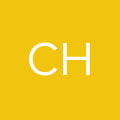


**You**

1. Please translate to Spanish: "Will a living donor have trouble getting health insurance or life insurance after they donate? Being a living donor may affect the ability to get health or life insurance. The transplant team can provide details."

ChatGPT

**ChatGPT**

Sure, here's the translation to Spanish:

"¿Tendrá un donante vivo problemas para obtener seguro de salud o de vida después de donar? Ser un donante vivo puede afectar la capacidad para obtener seguros de salud o de vida. El equipo de trasplante puede proporcionar detalles."


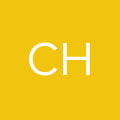


**You**

1. Please translate to Spanish: "How risky is living donor surgery? The surgery to donate a kidney is generally safe, with a 3 in 10,000 risk of death. The risks for living liver donation surgery are slightly higher, dependent on the amount of liver donated. More information about living donation risks is available on the United Network for Organ Sharing (UNOS) website, TransplantLiving.org. As with any surgery, living-liver donation can include complications such as bleeding, infection, bile duct problems, and blood clots, etc. Complications occur in about 16% of cases. Living donors are carefully evaluated prior to a living-donor liver transplant to ensure that risk is minimized. The estimated mortality is about 0.5% (5/1000), depending on which lobe (part) of the liver is donated."

ChatGPT

**ChatGPT**

Certainly, here's the translation to Spanish:

"¿Qué tan riesgosa es la cirugía de donante vivo? La cirugía para donar un riñón generalmente es segura, con un riesgo de muerte de 3 en 10,000. Los riesgos de la cirugía de donación de hígado en vida son ligeramente más altos, dependiendo de la cantidad de hígado donado. Más información sobre los riesgos de la donación en vida está disponible en el sitio web de la Red Unida para la Compartición de Órganos (UNOS), TransplantLiving.org. Como con cualquier cirugía, la donación de hígado en vida puede incluir complicaciones como sangrado, infección, problemas en los conductos biliares y coágulos de sangre, etc. Las complicaciones ocurren en aproximadamente el 16% de los casos. Los donantes vivos son cuidadosamente evaluados antes de un trasplante de hígado de donante vivo para asegurar que el riesgo sea minimizado. La mortalidad estimada es de aproximadamente 0.5% (5/1000), dependiendo de qué lóbulo (parte) del hígado se done."


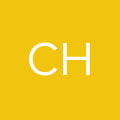


**You**

1. Please translate to Spanish: "Can adults over age 50 donate? Yes, donors must be healthy and can donate primarily on a basis of health. Some transplant hospitals may have age restrictions."

2 / 2

ChatGPT

**ChatGPT**

Sure, here's the translation to Spanish:

"¿Pueden los adultos mayores de 50 años donar? Sí, los donantes deben estar saludables y pueden donar principalmente en base a su salud. Algunos hospitales de trasplante pueden tener restricciones de edad."


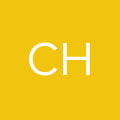


**You**

1. Please translate to Spanish: "Can members of the LGBTQ+ community be living donors? There are no restrictions based on a potential donor’s LGBTQ+ identification. All potential living donors undergo a thorough medical evaluation to determine if donation is safe."

ChatGPT

**ChatGPT**

Certainly, here's the translation to Spanish:

"¿Pueden los miembros de la comunidad LGBTQ+ ser donantes vivos? No hay restricciones basadas en la identificación LGBTQ+ de un posible donante. Todos los posibles donantes vivos se someten a una evaluación médica exhaustiva para determinar si la donación es segura."


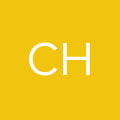


**You**

1. Please translate to Spanish: "Can people living with HIV be living donors? The HIV Organ Policy Equity Act (HOPE) Act provides donation and transplantation opportunities for people living with HIV with minimal additional testing at specific centers. Refer to the OPTN HOPE (HIV Organ Policy Equity) Act page for more information."

ChatGPT

**ChatGPT**

Sure, here's the translation to Spanish:

"¿Pueden las personas que viven con VIH ser donantes vivos? La Ley de Equidad de Política de Órganos VIH (HOPE Act) proporciona oportunidades de donación y trasplante para personas que viven con VIH con pruebas adicionales mínimas en centros específicos. Consulte la página del Acta HOPE (HIV Organ Policy Equity) de la OPTN para obtener más información."


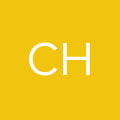


**You**

1. Please translate to Spanish: "Are people with tattoos able to be living donors? All potential living donors undergo a thorough medical evaluation to determine if donation is safe for both the donor and recipient."

ChatGPT

**ChatGPT**

Certainly, here's the translation to Spanish:

"¿Pueden las personas con tatuajes ser donantes vivos? Todos los posibles donantes vivos se someten a una evaluación médica exhaustiva para determinar si la donación es segura tanto para el donante como para el receptor."


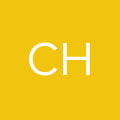


**You**

1. Please translate to Spanish: "What happens if a living donor changes their mind? Being a living donor is completely voluntary. The donor can delay or stop the process at any time. Their reasons for choosing to not donate will remain confidential and will not be shared with the recipient or anyone else."

ChatGPT

**ChatGPT**

Sure, here's the translation to Spanish:

"¿Qué sucede si un donante vivo cambia de opinión? Ser un donante vivo es completamente voluntario. El donante puede retrasar o detener el proceso en cualquier momento. Sus razones para elegir no donar permanecerán confidenciales y no se compartirán con el receptor ni con nadie más."


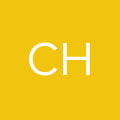


**You**

1. Please translate to Spanish: "Do living donors ever share their experiences and what are some resources? Yes, 97% of living donors say they would donate again and are excited to share their experiences. Donate Life WELD (We Encourage Living Donation), a program of Donate Life America, provides a forum for living donors to share their stories with members of their community. To learn more about DL/WELD, contact DL/WELD coordinator. View stories from living donors and recipients."

ChatGPT

**ChatGPT**

Certainly, here's the translation to Spanish:

"¿Comparten los donantes vivos alguna vez sus experiencias y cuáles son algunos recursos? Sí, el 97% de los donantes vivos dicen que donarían de nuevo y están emocionados de compartir sus experiencias. Donate Life WELD (We Encourage Living Donation), un programa de Donate Life America, ofrece un foro para que los donantes vivos compartan sus historias con miembros de su comunidad. Para aprender más sobre DL/WELD, contacte al coordinador de DL/WELD. Vea historias de donantes vivos y receptores."


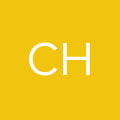


**You**

1. Please translate to Spanish: "How would a potential living donor start the process if they want to donate to a specific person? Contacting the transplant hospital of the person in need of the transplant is the first step. Many hospitals have a form on their website potential donors can submit to begin the process or they can call the transplant hospital and ask to speak with the living donor coordinator for the organ they wish to donate."

ChatGPT

**ChatGPT**

Sure, here's the translation to Spanish:

"¿Cómo empezaría el proceso un posible donante vivo si quiere donar a una persona específica? Contactar al hospital de trasplante de la persona que necesita el trasplante es el primer paso. Muchos hospitales tienen un formulario en su sitio web que los posibles donantes pueden enviar para comenzar el proceso o pueden llamar al hospital de trasplante y pedir hablar con el coordinador de donantes vivos para el órgano que desean donar."


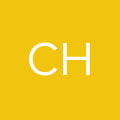


**You**

1. Please translate to Spanish: "What if the intended living kidney donor and intended recipient are not a match? Begin by talking to the transplant hospital where the intended recipient is listed to see if they participate in paired donation. The OPTN Kidney Paired Donation Program facilitates paired donation between transplant hospitals."

ChatGPT

**ChatGPT**

Certainly, here's the translation to Spanish:

"¿Qué pasa si el donante vivo de riñón previsto y el receptor previsto no son compatibles? Comience hablando con el hospital de trasplante donde está inscrito el receptor previsto para ver si participan en la donación apareada. El Programa de Donación de Riñón Apareado de la OPTN facilita la donación apareada entre hospitales de trasplante."


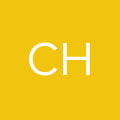


**You**

1. Please translate to Spanish: "What is the first step for a potential living donor who does not have a specific recipient in mind? Please refer to the Organ Procurement and Transplantation Network (OPTN) member list of all U.S. transplant hospitals. You may also want to consider being part of a living kidney donor paired exchange or chain. (see above)"

ChatGPT

**ChatGPT**

Sure, here's the translation to Spanish:

"¿Cuál es el primer paso para un posible donante vivo que no tiene en mente a un receptor específico? Consulte la lista de miembros de la Red de Obtención y Trasplante de Órganos (OPTN) de todos los hospitales de trasplantes de EE. UU. También puede considerar formar parte de un intercambio o cadena de donantes de riñón vivos apareados. (ver arriba)"
